# HGF/SF Increases Number of Skin Melanocytes but Does Not Alter Quality or Quantity of Follicular Melanogenesis

**DOI:** 10.1371/journal.pone.0074883

**Published:** 2013-11-06

**Authors:** Agnieszka Wolnicka-Glubisz, Anna Pecio, Dagmara Podkowa, Przemyslaw Mieszko Plonka, Maja Grabacka

**Affiliations:** 1 Department of Biophysics, Faculty of Biochemistry, Biophysics and Biotechnology, Jagiellonian University, Kraków, Poland; 2 Department of Comparative Anatomy, Institute of Zoology, Jagiellonian University, Kraków, Poland; 3 Department of Food Biotechnology, University of Agriculture, Kraków, Poland; University of Tennessee, United States of America

## Abstract

Melanins are an important factor determining the vulnerability of mammalian skin to UV radiation and thus to UV-induced skin cancers. Transgenic mice overexpressing hepatocyte growth factor/scatter factor (HGF/SF) have extra-follicular dermal melanocytes, notably in the papillary upper dermis, and are susceptible to UV-induced melanoma. Pigmented HGF/SF neonatal mice are more susceptible than albino HGF/SF animals to UVA -induced melanoma, indicating an involvement of melanin in melanoma formation. This raises the question of the effect of transgenic HGF/SF on melanization. We developed a methodology to accurately quantitate both the production of melanin and the efficiency of melanogenesis in normal, and HGF/SF transgenic mice *in vivo*. Skin and hair shafts of 5 day old and adult (3 week old) C57BL/6-HGF/SF and corresponding C57BL/6 wild type mice were investigated by electron paramagnetic resonance spectroscopy (EPR) to quantitate melanin, by transmission electron microscopy (TEM) for the presence of melanosomes, and by standard histology and by Western blotting and zymography to determine the expression and activity of melanogenesis-related proteins. Eumelanin but no phaeomelanin was detected in transgenic C57BL/6-HGF and C57BL/6 wild type mice. Transgenic HGF/SF overexpression did not change the type of melanin produced in the skin or hair, did not affect the terminal content of melanin production in standard samples of hair and did not influence hair cycle/morphogenesis-related changes in skin thickness. No melanocytes were found in the epidermis and no melanosomes were found in epidermal keratinocytes. HGF/SF transgenic mice thus lack the epidermal melanin UV-protection found in constitutively dark human skin. We conclude that melanocytes in the HGF/SF transgenic mouse, particularly in the papillary dermis, are vulnerable to UVA which interacts with eumelanin but not phaeomelanin to induce melanoma.

## Introduction

In contrast to humans, in the mouse trunk skin melanocytes are localized exclusively in the hair follicles [1-3]. Therefore, most of the animal melanomas, as potentially derived from follicular melanocytes, cannot directly be translated to human disease. Among animal models of melanoma, the HGF/SF transgenic mouse is of particular interest, as it possesses additional copies of HGF/SF gene that lead to extrafollicular localization of melanocytes [4,5]. These extrafollicular melanocytes are prone to UV-induced malignant transformation when the neonatal HGF/SF transgenic mouse is exposed to UV radiation [5,6]. Nevertheless, in contrast to epidemiologic studies showing that constitutively dark human skin is less sensitive to development of UV-induced skin cancer, including melanoma, than the fair phototype, in the HGF/SF transgenic mice, the effect is reversed and black pigmented HGF/SF transgenics are more susceptible to melanoma, particularly to UVA-induced melanoma although the rate of UVB-induced melanoma formation was not altered by the presence of melanin [ 6]. The distribution of melanin in HGF/SF transgenic mouse skin is different from that in humans, i.e. there is little melanin in the epidermis of mice compared to constitutively dark humans where melanin is abundant in the epidermis. Yamazaki et al. [7] demonstrated that epidermal melanin prevented UVB-induced DNA damage and the related UV carcinogenesis in another model of transgenic mice, namely the cross of *xeroderma pigmentosum* A – deficient and stem-cell-factor-positive (*Xpa* ^*-/-*^ *, SCF* ^*+/+*^-transgenic) mice on a mixed genetic background (CBA, C57BL/6, CD-1, and “hairless” Hos/HR-1). These black, hairless hybrids revealed decreased vulnerability towards development of UVB-induced skin cancer [7]. However, these animals did not develop melanoma. In contrast, black transgenic HGF/SF mice developed melanomas more rapidly and had more melanoma lesions per recipient than albino HGF/SF transgenics, and UVA induced melanoma only in the black animals [5,6]. So the actual role of melanin in the UV-induced development of melanoma remains still an open problem. The question therefore arises if the melanin in HGF/SF transgenic mice is different in composition from human melanin.

Melanins are polymorphous and multifunctional biopolymers, represented by eumelanin, pheomelanin, and mixed melanin pigment [2,8]. They are the end-products of complex multistep transformations of L-phenylalanine and/or L-tyrosine with or without participation of L-cysteine and/or glutathione [2,8,9]. They are responsible for the formation of various coat color patterns which are important for camouflage, mimicry and signaling. Melanins are commonly considered as versatile photoprotectors, mainly against UV radiation, and they prevent radiation-induced free-radical damage [2]. But, on the other hand, there are numerous reports showing the toxicity of their precursors and the products of their degradation, including photodegradation [2,9-12]. Consequently, their actual role in carcinogenesis and in the induction or protection of melanoma is controversial [13]. In particular, it may depend on the kind of pigment produced (eu- or pheomelanin), as well as on the intensity of melanogenesis.

In the present paper we examine the differences in the intensity and quality of melanogenesis as a result of HGF/SF overexpression in the skin of neonatal and adult C57BL/6 mice (wild-type or transgenic HGF/SF). It is important to note the possibility that HGF/SF exerts some additional indirect effects which may have impact on the net melanin content in the skin. Both HGF/SF and its receptor c-Met are involved in the regulation of hair cycle in the mouse [14]. It was shown that HGF/SF stimulates the hair follicle growth of mouse vibrissae and human hair in an organ culture system [15]. Neonatal 3 day old HGF/SF transgenic mice compared with matched wild type animals had twice as many developed hair follicles (HF) and a doubled speed of HF morphogenesis and HGF/SF overexpression retarded catagen development [14]. In addition to these regenerative effects, anti-apoptotic and anti-inflammatory roles of HGF have been widely demonstrated *in vitro* and *in vivo* [16,17]. Therefore, the main questions addressed in the present paper focus on the quality and quantity of melanin produced in the HGF/SF transgenic mice. First, we employed spectroscopy of the electron paramagnetic (spin) resonance, EPR (ESR), and transmission electron microscopy (TEM) to check the morphology of melanocytes and melanosomes. The net efficiency of melanogenesis was examined on the level of the melanin product present in the hair shafts and in the skin, appropriately normalized to control for other hair-follicle, hair-morphogenesis, and hair-cycle-related effects of HGF/SF. In parallel, the protein levels of the main melanogenic enzymes – tyrosinase and dopachrome tautomerase (tyrosinase-related protein 2, Dct) [2,9], and the microphtalmia transcription factor (MITF), the key transcription factor involved in pigmentation [18] were assessed by immunoblotting. Additionally the activity of tyrosinase was determined by gel zymography. 

## Materials and Methods

### Animals

Hair and trunk samples of C57BL/6, C57BL/6-HGF/SF, C57BL/6-c, C57BL/6-Mc1r^e/e^, C57BL/6J-*A* ^y/a^ were a kind gift of professor Frances Noonan (George Washington University, DC, USA). The samples were collected at The George Washington University as part of a protocol which employed HGF transgenic and corresponding wild-type animals on various genetic backgrounds to understand the role of UV radiation in melanoma. The studies were reviewed and approved by The Institutional Animal Care and Use Committee of The George Washington University according to the PHS (Public Health Service, USA) Policy on Humane Care and Use of Laboratory Animals, The Guide for the Care and Use of Laboratory Animals, National Research Council of the National Academy of Science, USA and the American Veterinary Medical Association Guidelines for Anesthesia. The approved protocol number was 001-1. The George Washington University Animal Research Facility operates under Assurance A3205-1 from the Office of Laboratory Animal Welfare, National Institutes of Health and is an AAALAC (Association for Assessment and Accreditation of Laboratory Animal Care) accredited facility. Findings under this approval using samples from C57BL/6, C57BL/6-HGF, C57BL/6-*c*, C57BL/6-c-HGF animals have been published [6] and findings from samples from C57BL/6-Mc1r ^e/e^ mice approved under this protocol were published in [19]. Data on samples from C57BL/6J-A^y/a^ mice approved under this protocol has not been published.

### Electron microscopy and morphometry

Skin cut in small pieces was fixed in modified Karnovsky's fixative containing 2% paraformaldehyde, 2.5 % glutaraldehyde in 0.1 M cacodylate buffer at pH = 7.2 for 2 h and processed according to the previously described method [20]. The grids with ultrathin sections were next examined under a JEOL JEM-100Sx transmission electron microscope (Tokyo, Japan). Melanosome size was expressed as the area and perimeter, and was measured using MigraNew4 image analysis software (Mr R.Tokarski from the Department of Cell Biology, UJ [21]).

### EPR spectroscopy

Due to the presence of semiquinone-like units in the melanin structure these polymers have strong paramagnetic properties [2,20]. Eumelanins and pheomelanin differ in chemical composition, as well as in their physical properties [2,22]. The EPR signal of eumelanin is a slightly asymmetric singlet of g = 2.004 (typical of free-radical paramagnetic centers). For pheomelanins this EPR signal is additionally splitted due to hyperfine interactions of the unpaired electron with the nucleus of ^14^N [23,24]. This splitting is a specific feature of pheomelanin, and the contribution of the latter can be expressed as the ratio of the half-amplitude of the central line (present in the signals of both types of polymers) to the low-field component (characteristic for pheomelanin). This parameter is a convenient tool that can distinguish between the types of melanin and estimate its content in the tissue [23].

DOPA and cysteinyldopa melanin, the synthetic equivalents for eumelanin and an eu-pheomelanin co-polymer, were synthesized as reported [20]. The content and type of melanin were determined using an X-band (ca. 9.2 GHz) “Varian E 3” EPR spectrometer with 100 kHz modulation and a rectangular TE 102 resonant cavity (Varian, Inc., Palo Alto, CA, USA) at 77 K or ambient temperature (murine hair), field 3269±50 Gs, modulation amplitude 0.5 Gs (qualitative assay) or 5 Gs (quantitative assay), time constant 100 ms, microwave power 0.25 or 1 mW (respectively), receiver gain from 6200 (DOPA melanin) – to 1250000 (albino fur), scan time 180-200 s, resolution 1024 points. 

Frozen skin samples, capillaries with powdered synthetic melanins, or hair were put into a quartz finger dewar and placed in the cavity, to preserve constant geometry of the samples and the measurement conditions [24]. All the signals were expressed as the peak-to-peak amplitudes and verified using the integral intensities of the signals. The contribution of pheomelanin was expressed as the a/b parameter, i.e. by the ratio of hyperfine components [2,23,24]. To analyze data quantitatively, means ± SD of at least 3-4 independent samples were calculated. The signal intensities were additionally normalized for a constant gain (400 000) and constant sample mass (100 mg). The intensities of the skin samples were further normalized per a constant skin area (constant reverse-thickness, see [24-26]) and a constant hair follicle number in a skin area unit. A small powder sample of 1,1-diphenyl-2-picrylhydrazyl (DPPH) served as a marker for the position of the free radical signal (g=2.0037). The statistical significance of the differences in signal intensities, a/b parameters, and linewidths were tested by the unpaired two–tailed Student *t*-test, and accepted for P<0.05.

#### Histology

Skin pieces from 5 and 21 days old C57BL/6 and C57/BL-6-HGF/SF mouse were fixed in formalin, then embedded in paraffin, cut into 5 μm thick sections, stained routinely with hematoxylin and eosin, and examined under a microscope (Nikon Eclipse E-100, Tokyo, Japan).

#### Immunoblotting

Tyrosinase, Dct and MITF proteins were detected in the mouse skin tissue by immunoblotting. Briefly, murine skin tissue samples were chopped, washed in sterile PBS, and homogenized in a glass-teflon homogenizer in the TNN buffer [50 mmol/L Tris-HCl (pH 7.5), 150 mmol/L NaCl, 0.5% NP40, protease inhibitors without EDTA (Roche)]. Aliquots of 50 µg of protein extracts were separated in 10 % polyacrylamide gels in SDS-PAGE and transferred onto nitrocellulose membranes (Thermo Fisher Scientific Inc., Waltham, MA, USA). The resulting blots were blocked in 5% nonfat milk in Tris-buffered saline with 0.5% Tween 20 and probed with the primary antibodies: rabbit polyclonal anti-PEP7 detecting tyrosinase and anti-PEP8 detecting Dct (kindly provided by Dr. Vincent Hearing, NIH, Bethesda, USA), anti–MITF-CT mouse monoclonal (a generous gift from Dr. Heinz Arnheiter, NIH, Bethesda, USA) and Grb-2 (mouse monoclonal, BD Transduction Laboratories, BD, Franklin Lakes, NJ USA), as a reference protein to check equal loading. Following the incubation with the secondary HRP-conjugated antibodies, chemiluminescent signal detection was performed. Semi-quantitative densitometric analysis of immunoblots was performed with ImageJ software. 

#### Tyrosinase activity

Tyrosinase activity was determined by zymography according to Jimenez-Cervantes et al. [27]. Briefly, aliquots of tissue extracts (100 μg) were separated by native electrophoresis. The gels were washed 2×10 min in acidifying buffer (50 mM sodium phosphate buffer, pH 6.0) and transferred to the developing solution (10 mM sodium phosphate buffer, pH 6.8, 1.5 mM L-DOPA, 4 mM MBTH) and incubated in 37°C until the reddish-brown bands appeared (approximately 2 h).

## Results and Discussion

### Hair cycle stages in the investigated skin

**Figure 1:**
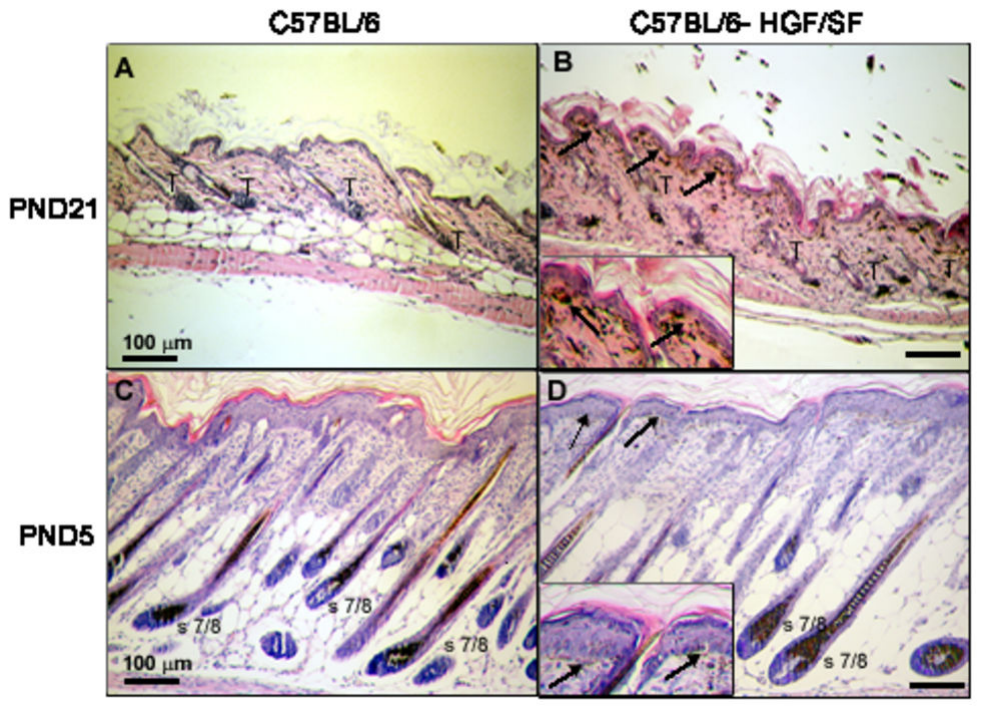
Haematoxylin and eosin staining of mouse skin of different age and hair cycle. (A,C) C57BL/6. (B,D) C57BL/6-HGF/SF. (A-B) PND21 (postnatal day 21). (C-D)- PND5 (postnatal day 5). Arrows – extrafollicular melanocytes; T- telogen hair follicle; s 7/8- stage 7/8 of hair follicle morphogenesis. Scale bar - 100 µm.

In the normal trunk skin of adult mice mature melanocytes are present only in the hair follicles [2,28], and only in anagen which is the stage of hair cycle when the active growth and pigmentation of hair takes place [29,30]. Intensity of follicular melanogenesis in C57BL/6 mice in anagen VI is very high [25], and the follicular production of melanin would be a high “background” for generation of the pigment by the extrafollicular melanocytes in the transgenic HGF/SF animals. So far it seems impossible to measure *in vivo* separately follicular and extrafollicular melanin. Therefore, to effectively compare the process in the skin of the wild and the transgenic animals, the best stage of the hair cycle is telogen, i.e. the “resting” stage [26], when no melanin is produced in the hair follicle [1]. In Figures 1A and 1B we show the histological skin sections of mature animals of both genotypes C57BL/6 and C57BL/6-HGF, which were examined in this study. Both groups contain only telogen hair follicles [31], confirmed by a relatively low thickness of the skin. In the HGF/SF transgenic group, in the dermal layer of skin active extrafollicular melanocytes producing melanin can be noticed. 

The embryonic development of hair follicles starts prenatally, but it continues after birth. The process can be divided into 8 stages [32] and it is in progress at the very moment of birth, so normally no “telogen” newborn mice exist. Moreover, the number of the extrafollicular melanocytes in the transgenic HGF/SF mice also increases gradually [6], and to make it able to compare the process of their melanogenesis with mature skin, 5-days-old newborn mice were used. At this time point their hair follicles reveal stage7/8 of morphogenesis [32] which is quite analogous to anagen VI of the postnatal hair cycle [31]. The skin of wild-type C57BL/6 and transgenic C57BL/6 HGF/SF mice contains hair follicles in this stage of morphogenesis, which can be further confirmed by a higher thickness of skin, as compared to the telogen skin. Both phenomena are shown in Figures 1C and 1D, and additionally, the presence of extrafollicular melanocytes in the transgenic animals (Figures 1B and 1D, insets) can be noticed.

### Dermal melanocytes in HGF/SF transgenic mouse skin contain mature melanosomes

**Figure 2:**
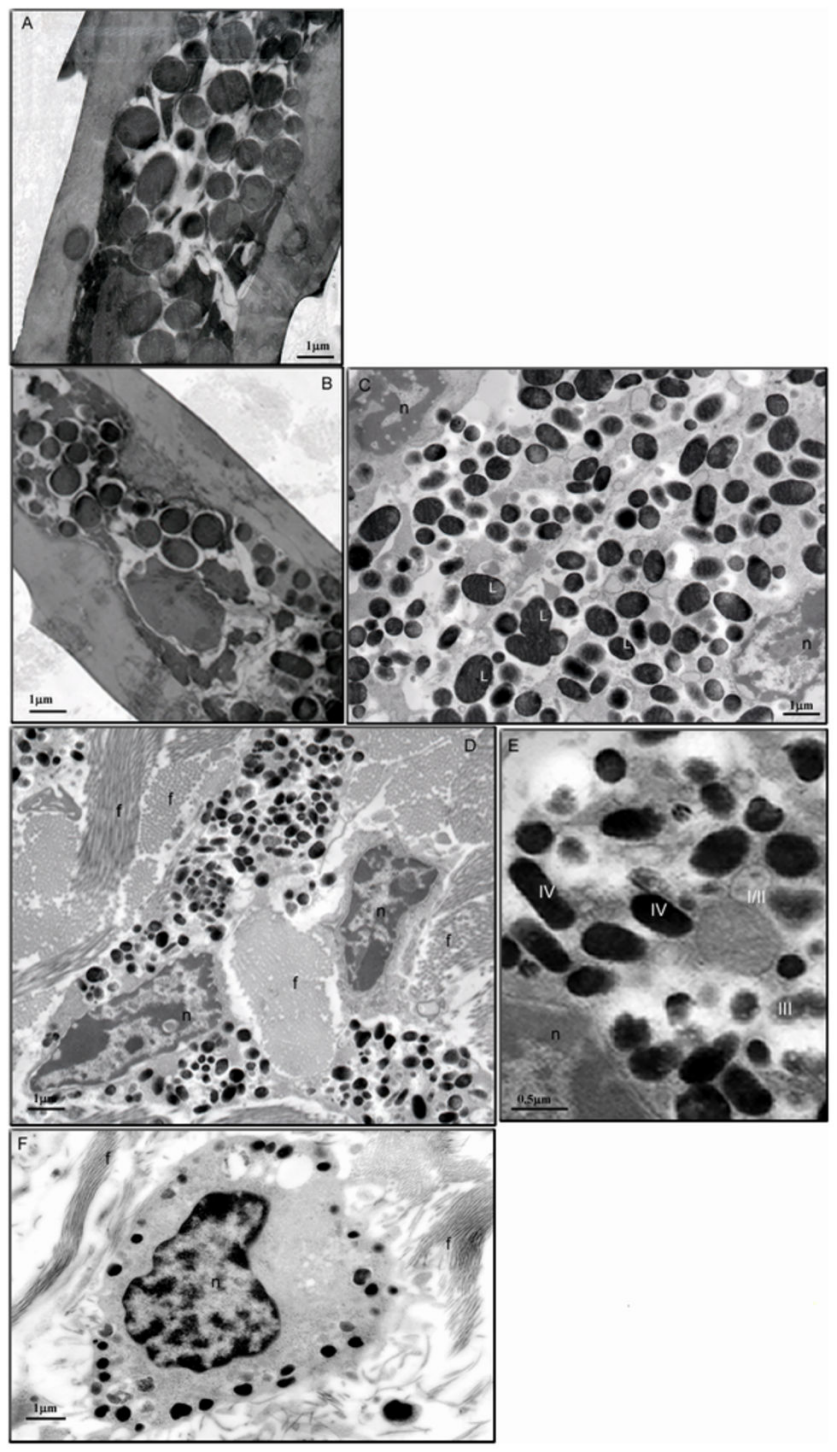
TEM pictures of melanocytes in mouse hair and skin. (A-B) Melanocytes in adult hair shaft; (A) wild-type C57BL/6 (B) C57BL/6-HGF/SF. (C-F) Melanocytes in C57BL/6-HGF/SF transgenic mouse; (C) hair bulb. (D-E) adult skin. (F). PND5 skin. f- fiber, n-nucleus, l- lamellar structure. Melanosome differentiation stages I-IV.

In this study we confirmed that extrafollicular melanocytes are present in the dermis of C57BL/6-HGF/SF adult and neonatal mouse skin (Figures 1 and 2) but not in wild type animals. Extrafollicular melanocytes were found only in the dermis in the stratum papillare and subpapillare. There were no melanocytes in the epidermis of C57BL/6-HGF/SF of both adult and neonate and we did not find melanosomes in epidermal keratinocytes. Dermal melanocytes in adult mouse skin contain about 64 ± 29 melanosomes per cell (Figure 2D), while in 5-day-old pups about 33 ± 13 melanosomes per cell (Figure 2F) can be found. About 70% of melanosomes are ellipsoidal, which confirms their eumelanosome-like character, although some melanosomes of earlier stages (I-III) can be found, too (Figure 2E). As the orientation of melanosomes in hair is random, the oval shape of some sections may be attributed to the direction of the cut (transversal instead of longitudinal). In some of the organellae, lamellar ultrastructure can be noticed (Figure 2C), while there are no round melanosomes with vacuolized or irregular ultrastructure typical of pheomelanosomes. 

The area of melanosomes in adult HGF/SF transgenic mouse skin varied between 0.01-0.22 µm^2^ (the mean 0.072 ± 0.035µm^2^). In human skin the size of melanosomes is correlated with the skin phototype, reaching about 0. 0144 ± 0.0067 µm^2^ in the negroidal and 0.0094 ± 0.0048 μm^2^ in the Caucasian phenotype [33]. So, murine melanosomes in the C57BL/6-HGF/SF transgenic mouse are 4-7 times larger than human melanocytes. Furthermore, follicular melanosomes, of C57BL/6-HGF/SF mice have a 6 times larger area and 2.5 times larger perimeter than melanosomes in the extrafollicular melanocytes (Table 1). Similarly, in human Caucasian skin, melanosomes are 354 ± 35 nm long and 109 ± 33 nm broad but in hair follicles are 918 ± 10 nm long and 309 ± 24 nm broad, so they are 2.5 times larger than the epidermal melanosomes [34], in agreement with our present observations in the murine model. Neither the size nor the shape of hair shaft melanosomes of transgenic C57BL/6-HGF/SF mice differ from the ones of the wild-type C57BL/6 (Figure 2 A,B and Table 1). Morphometric analysis of dermal and follicular melanocytes revealed that they contain mostly mature eumelanosomes regardless of animal age. All these data strongly suggest that that the type of melanin produced in the skin of transgenic C57BL/6-HGF/SF mice is eumelanin. 

**Table 1:** Morphometric parameters of melanosomes in adult and pup mouse.

	**C57BL/6**	**C57BL/6-HGF/SF**			
**age**	**adult**				**PND5**
	hair shaft (n=59)	hair shaft (n=156)	hair bulb (n=640)	skin (n=823; N=69±29)	skin (n=139; N=33±13)
**area (μm^2^)**					
Min	0.09	0.04	0.02	0.01	0.01
Max	0.75	1.67	1.54	0.22	0.31
mean ± SD p	0.4±0.25 -	0.41±0.3 0.91	0.29*±0.2 9.659x10^-9^	0.072*±0.035 0	0.062**± 0.041 0.000285
**perimeter (μm)**					
Min	1.7	0.69	0.63	0.52	0.63
Max	7.38	7.59	8.65	3.7	3.5
mean ±SD p	2.98 ± 1.29 -	2.85±1.34 0.527	2.5*±1.14 0.00129	1.18*±0.41 0	1.22**±0.50 0

n- the number of melanosomes analyzed; N- the number of melanosome per melanocyte;

* statistically significant difference from adult hair shaft melanosomes (p < 0.05);

** statistically significant difference from adult skin melanosomes (p< 0.01).

### HGF/SF does not affect the type of melanin in dermal and follicular melanocytes

**Figure 3:**
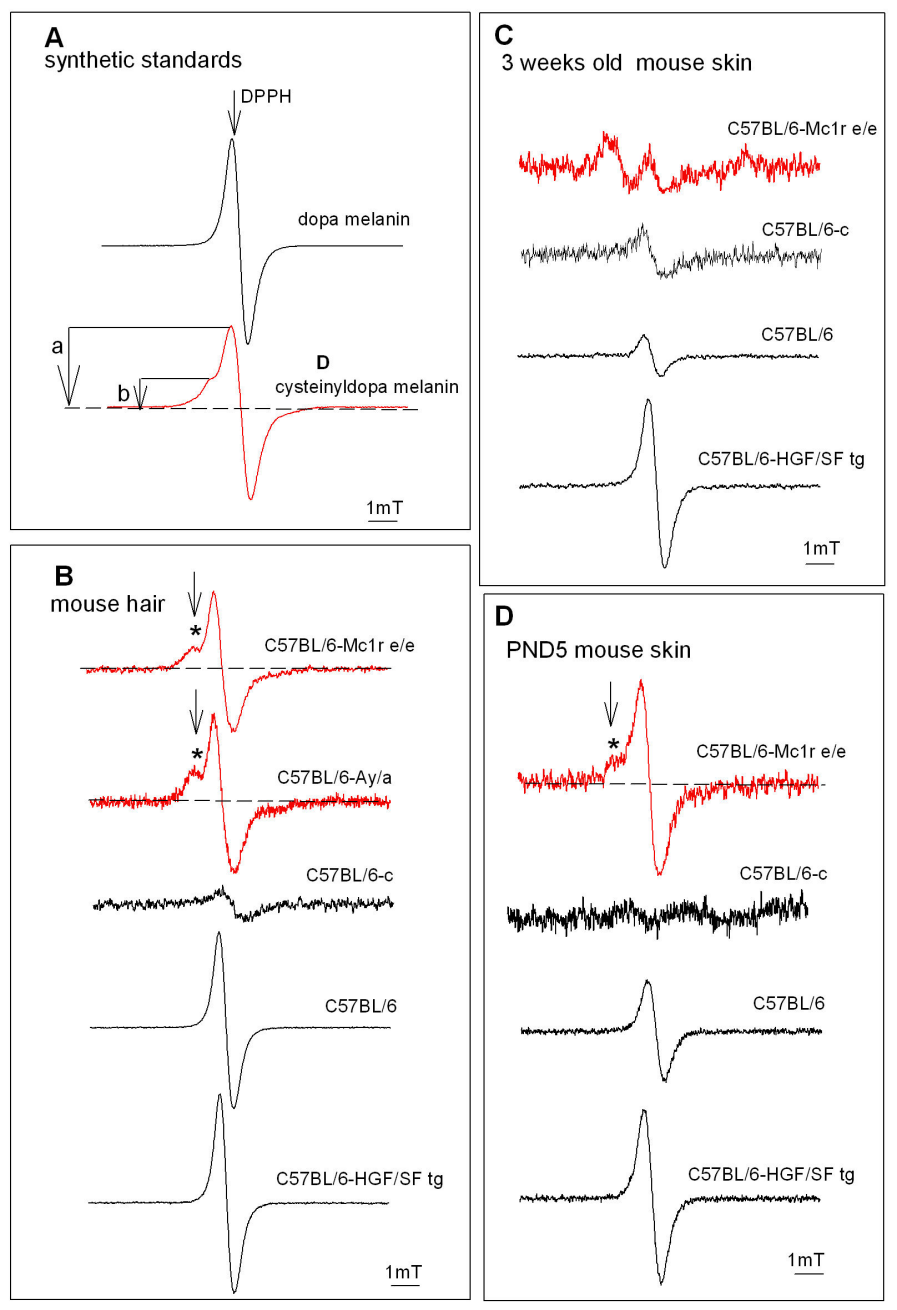
EPR spectra of the investigated materials. DPPH (1,1-diphenyl-2-picrylhydrazyl) – marker for the position of the free radical signal (g=2.0037). (A) Dopa melanin and cysteinyl dopa melanin. (B) Hair of adult mouse (20 mg). (C) Melanin in skin (100 mg) of adult. (D). PND5 mouse skin. *- indicates the low field component of the splitting, used to calculate the a/b parameter (A). C57BL/6 and C57BL/6-HGF skin is compared to samples known for its content of pheomelanin, and often used as a pheomelanotic standard: hair of yellow C57BL/6-A^y/a^ and C57BL/6- Mc1r^e/e^ mice, and with amelanotic C57BL/6-*c*. Note that the pigment contained in the skin and in the hair is not pure pheomelanin, but a co-polymer with high contribution of pheomelanin. Also the used synthetic cysteinyldopa-melanin, besides semiquinonimine centres, contains semiquinones typical of eumelanins, with no interacting ^14^N.

**Figure 4:**
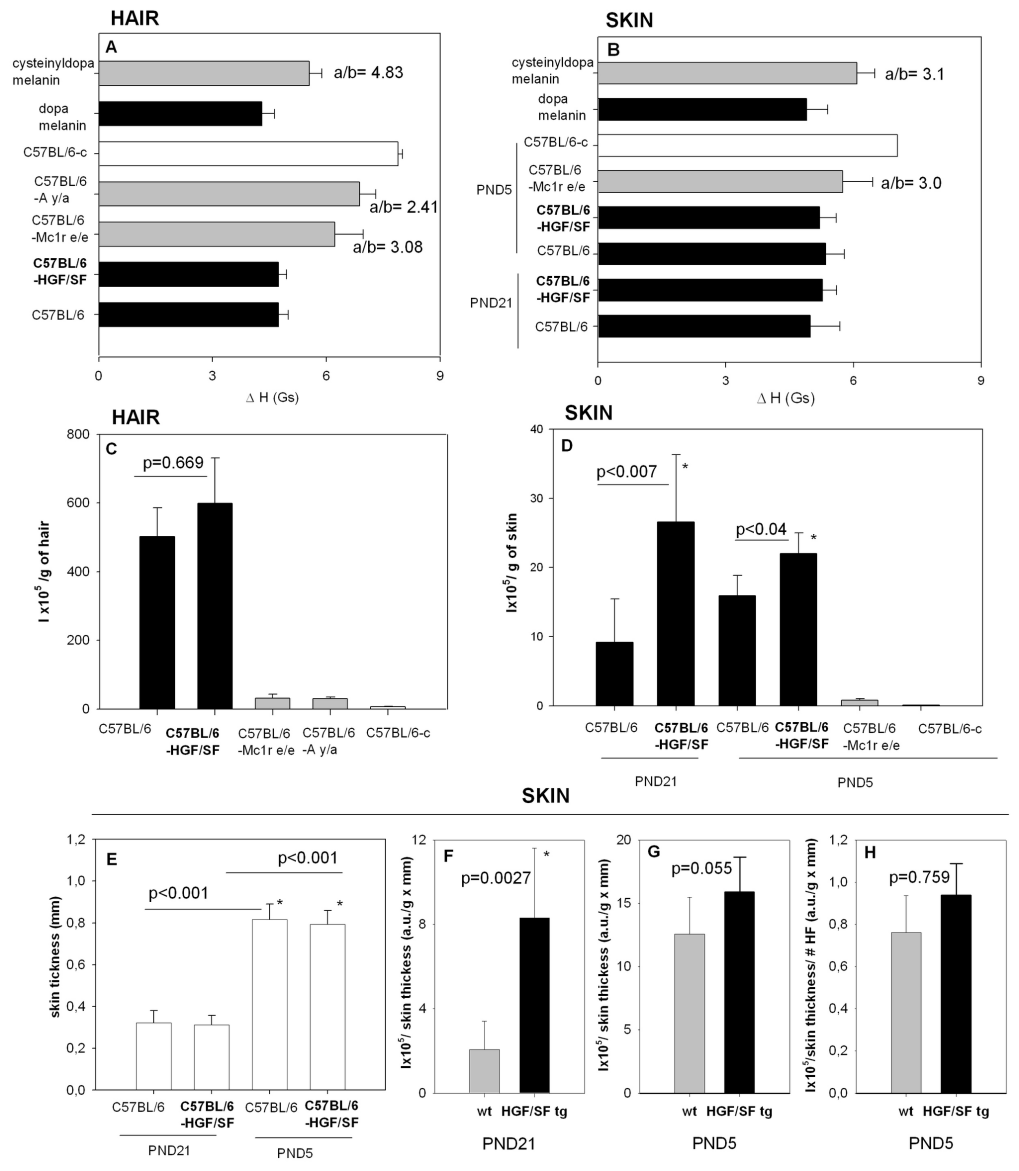
Quantitative analysis of the EPR measurements. (A-B) The EPR signals line widths of analyzed materials. (C-D) Amplitudes of the EPR signals normalized per tissue sample mass and amplification. (E) Skin thickness (mm). (F-G) Normalized amount of melanin per skin thickness. h- normalized amount of melanin per skin thickness and number of hair follicle (#HF) count per microscopic field 10×(MF). All the bars represent the means of 3-4 samples ± SD.

The EPR examination supported the conclusions drawn from the ultrastructural observations and confirmed the presence of eumelanin in both the wild type and the HGF/SF transgenic skin and hair of C57BL/6 mice (Figures 3 and 4A-B). We carried out a thorough analysis employing continuous wave (CW) EPR spectroscopy targeted at qualitative and quantitative comparisons. First of all, we have shown that the C57BL/6 HGF/SF transgenic mice produce in their hair follicles and in dermal melanocytes the same kind of melanin as the control mice. The EPR signals of C57BL/6 and C57BL/6-HGF/SF transgenic mouse skin, and hair shafts (Figure 3), regardless of mouse age, show a slightly asymmetric singlet of g = 2.004 (typical of free-radical paramagnetic centers) and which is characteristic of eumelanin [22,35]. The materials gave no EPR signals with hyperfine structure characteristic of pheomelanin and, importantly, the linewidths of the EPR signals (for C57BL/6 and C57BL/6-HGF/SF ) were very similar, arguing further against pheomelanin, the EPR signals for which tend to be wider than those of eumelanin [24,36]. In conclusion, the EPR analysis did not reveal any noticeable proportion of pheomelanin, either in normal C57BL/6, or in transgenic C57BL/6 HGF/SF hair or skin, while phaeomelanin was readily detected in known pheomelanic mice phenotypes (Figures 3 and 4 A-B), i.e. hair of C57BL/6-Mc1r ^e/e^ and C57BL/6-*A* ^y/a^ mice, the C57BL/6-Mc1r ^e/e^ neonatal mouse.

In summary, the overexpression of HGF/SF in the skin, both in the dermal and follicular melanocytes does not switch the type of the produced melanin towards pheomelanin. This is an important observation reported here for the first time*, -* as pheomelanin and in particular – its products of UV degradation seem more toxic and, perhaps, carcinogenic, than eumelanin [2,10-12]. Recently it was show that pheomelanin could cause melanoma in the B-RAF mouse model of melanoma in the absence of UV [37]. Our data however indicate that in the HGF mouse model the increased induction of melanoma in pigmented animals [6] is not due to pheomelanin.

### HGF/SF does not affect the terminal deposition of melanin in hair shafts

Both follicular and dermal melanocytes contribute to the total amount of melanin produced by a given fragment of skin. In the hair follicles, the mature melanin is deposited in the hair shafts in parallel to their elongation during anagen or morphogenesis, and it makes them the best material to compare the type and amount of melanin synthesized by follicular melanocytes, representing the terminal product of the pigmentation process – the pigmented hair shaft [24]. Although in the C56BL/6 HGF/SF transgenic mice the eumelanin level in the skin is higher as expected, the level of melanin in hair remains similar to that of the wild-type control (Figure 4C). The EPR analysis is simple and unambiguous, and the EPR signal amplitude or integral intensity is directly correlated with the amount of melanin in a mass unit of hair, thus representing the ultimate melanogenic activity of follicular melanocytes, which in the transgenic animals is seen to remain unaltered.

This strongly suggests that HGF/SF does not influence the efficiency or the mechanism of the melanocyte melanin production, but affects only the number and, probably, localization of the melanin-generating cells. Since the wild type mouse has no extrafollicular melanocytes the only possibility to estimate quantitatively melanogenesis in HGF/SF transgenic melanocytes is to make comparisons between follicular melanocytes.

### Quantitative EPR analysis of skin pigmentation

Quantitative analysis of melanogenesis in whole skin is more complicated than of dry hair shafts [24]. It requires a strict and careful regard of various factors which may influence the EPR signal intensity of a mass unit of pigmented skin. These factors include the stage of hair cycle or morphogenesis and the related skin thickness, as well as the number of hair follicles in the skin area unit.

The “crude” quantitative EPR results are presented in Figure 4D where we compared the skin melanization of neonatal 5 day old mice and adult mice. Since on day 5 mice do not posses a hair coat, we compared the neonatal skin with shaved adult skin. In this comparison it is readily noted that the transgenic animals reveal much stronger melanization, as expressed by the EPR signal amplitude of a mass unit of skin, both in adult and neonatal mice (Figure 4D). What calls for an explanation in the interpretation of this result, is that adult animals reveal stronger EPR signals than young animals, although the young skin was in stage 7/8 of morphogenesis, related to anagen VI, which is the stage of the strongest melanogenesis [32], while the skin of the adults was in telogen, i.e. in the hair cycle stage which should be completely devoid of follicular melanization [1,15].

The macroscopic parameter of skin which strongly depends on the stage of hair growth is skin thickness. During anagen VI skin becomes almost twice as thick as in telogen [25,38], and the same occurs in the final stages of hair morphogenesis [32]. This process is mainly due to edema, which results in an increase in the content of water and an apparent “dilution” of melanin. As we tried to keep geometry of measurement i.e. the volume of the samples constant, to obtain the samples of the telogen skin we had to use fragments of skin of much bigger areas (containing many more melanocytes) than of the stage 7/8 skin which is thicker [24,25]. To exactly compare the efficiency of follicular melanization, therefore, the results must be normalized per constant skin area, or per constant reverse-thickness, because for the same sample volume a bigger skin area means a lower thickness (see 24,25 for more explanations). Considering this fact, we obtained Figures 4F and G, which take into account the variable skin thickness between adults and neonates (Figure 4E). These two panels represent the amounts of melanin contained in the same area of skin of the adult (Figure 4F) or neonatal (Figure 4G) wild-type or HGF/SF transgenic mice, adjusted for skin thickness. Comparison of Panels F and G in Figure 4 shows that the wild type telogen skin of adult mice contains much lower amount of melanin (mainly in the remains of the shaved telogen hair shafts, see e.g. 2), than the stage 7/8 skin of the young animals. Importantly, parallel to the pigmentation studies, we found out that the overexpression of the HGF/SF gene does not affect the skin thickness (Figure 4E). We also conclude that the amount of melanin does not influence the skin thickness, which is readily seen in Figure 4E. This is another important observation, as in our earlier papers [26] we had questioned whether the hair-cycle-related parameter responsible for changes in the skin thickness is the melanin content. Here, we have two paired groups of mice in the same hair cycle stage (telogen or stage 7/8 of morphogenesis), of the same thickness, but with different amounts of melanin (wild type vs. HGF/SF transgenic). It is clear that it is the hair cycle phase, not the melanin content, that affects skin thickness (Figure 4E). This conclusion is valid both for the stage of the absence of hair follicle melanogenesis (adult telogen skin) and of the maximal intensity of melanogenesis (stage 7/8 of morphogenesis in neonatal skin). 

Another important factor affecting our results is the average number of hair follicles per skin area unit. In their paper, Lindner, Paus and colleagues [14], showed that the number of hair follicles significantly increases in the presence of transgenic HGF/SF. The higher intensity of the EPR signal in the neonatal transgenic HGF/SF mice than in the wild type control will, consequently, result not only from the presence of the extrafollicular, dermal melanocytes but also from the higher number of hair follicles which, in contrast to adult skin, are actively producing melanin. We therefore normalized the EPR results for neonatal skin not only by constant mass and constant skin area (Figure 4G), but also by the number of hair follicles (Figure 4H). For this calculation the EPR results were divided by the number of hair follicles visible in the same microscopic field (mf) under the same magnification. We found increased numbers of hair follicles in HGF transgenic skin (data not shown) in agreement with the cited authors [14]. The difference between the bars in Figure 4H representing the excess of melanin produced by the extrafollicular melanocytes, is insignificant, and smaller than the respective difference in Figure 4F, as the adult skin contains more extrafollicular melanocytes than the skin of the newborn mice ([6]; compare Figures 1B inset and 1D inset). 

It is important to note that the normalization by number of hair follicles is relevant only in the case of hair follicles actively producing melanin (stage 7/8 of morphogenesis, anagen III-VI of hair cycle). The number of hair follicles will not render any detectable effect on the melanin EPR signal in the telogen mice. Consequently, to compare the amounts of melanin between the same area of skin, and to quantify the effect of the overexpression of HGF/SF, the results must be additionally normalized per the same number of hair follicles only in the case of the young (i.e. stage 7/8) skin. The results (Figure 4H) represent the average amount of melanin per a single hair follicle. This tends to be higher for the transgenic mice, because they contain also additionally the extrafollicular melanocytes in the skin (Figure 1D) but is not significant. Figure 4G was prepared to make it possible to compare the melanogenic activity of the young and adult skin, which depends not only on the overexpression of HGF/SF, but also the hair cycle/development stage.

We conclude therefore that the increased EPR signal parameters detected in the skin of C57BL/6-HGF/SF adult mice (Figures 3 and 4E) compared to wild-type animals must represent the dermal (non follicular) melanocytes, which continue to produce melanin even in telogen. In the neonatal HGF/SF transgenic mice, where the number of dermal melanocytes is lower relative to pigment producing cells in the follicle, the normalized EPR signal is not significantly higher than in the wild-type. 

Further, in addition to the quantitative observations of the influence of HGF/SF on melanogenesis in the C57BL/6 murine skin, in this paper we have described procedures for normalization of the EPR results to enable comparison of various aspects of skin melanogenesis, similar to the studies on telogen previously published [26]. 

### Tyrosinase, Dct and tyrosinase activity in C57BL/6-HGF/SF transgenic mouse skin depends on the hair cycle and the presence of dermal melanocytes

**Figure 5:**
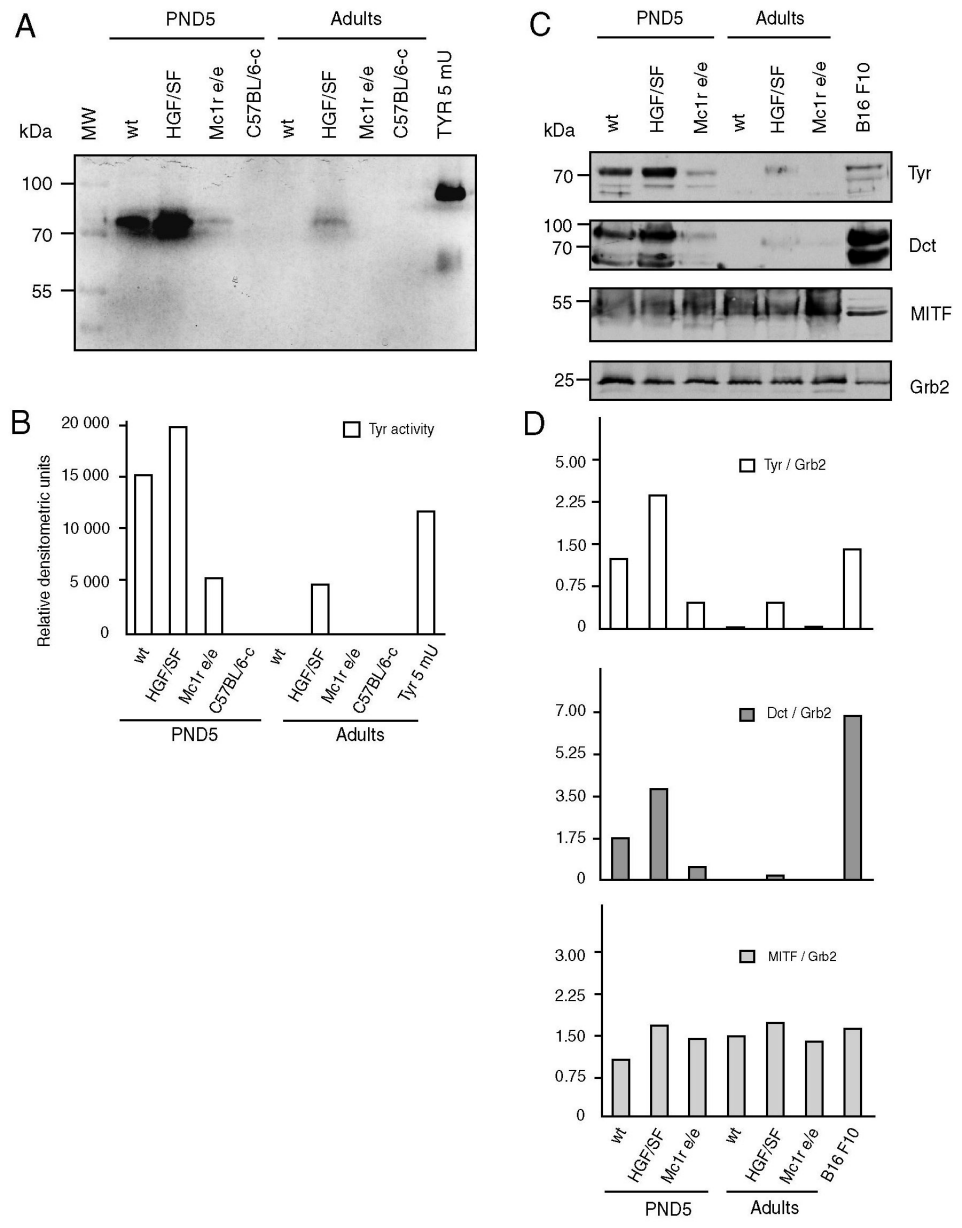
Tyrosine activity and expression of tyrosinase, Dct and MITF in PND5 and 3 weeks (adult) C57BL/6 mouse skin. (A) Zymography of tyrosinase activity. (B) Densimetric analysis of the zymogram, positive control-purified tyrosinase from *Agaricus* *bisposrus*, 5 mU (TYR 5 mU), negative control C57BL/6-*c*. (C) Immunoblotting. (D) Densitometric analysis of the bands – Tyrosinase (Tyr), DOPAchrome tautomerase (Dct) and MITF expression was normalized to a reference protein Grb-2, that served as a loading control. The Western blot experiments were performed on two different set of the samples, each set repeated twice. The figure shows the representative results.

Finally, we accompanied our EPR and morphologic data with a study on molecular factors influencing skin pigmentation, i.e. activity of tyrosinase (Figures 5A and 5B) and expression of tyrosinase, Dct and MITF, a regulator of melanogenesis (Figures 5C and 5D). Indeed – the expression and activity of tyrosinase (Figures 5B and 5D) and the expression of Dct (Figure 5D) is higher in the transgenic C57BL/6-HGF/SF mice than in the wild control. Tyr and Dct are present in the wild type young animals, because they are in stage 7/8 of follicular morphogenesis, but are absent in the wild type adult, which is in telogen. As expected, tyrosinase activity and expression of Tyr and Dct in the skin of yellow C57BL/6-Mc1r^e/e^ is weaker than in the wild type C57BL/6 animals, and is absent in the albino C57BL/6-*c* (Figures 5A and 5B). 

The results of the assay for the expression of MITF demand additional comment. This factor is key for regulation of the expression of melanogenesis-related enzymes [18], and one might expect its absence in the melanogenetically inactive materials, e.g. in the adult wild-type telogen skin (Figure 5). Nevertheless, it is present, but one has to consider that it is not an effector, melanogenesis-related molecule, but a crucially important transcription factor that regulates the expression, besides tyrosinase and Dct also of more than 25 genes in melanocytes [18]. It has emerged as an essential protein coordinating the melanocyte development, proliferation and survival, and also the assembly of the enzymatic and structural apparatus necessary for the melanin biosynthesis, and moreover, is active and necessary also for other skin cells [18]. 

The results shown in Figure 5 clearly show that the transgene (HGF/SF) does not specifically affect MITF expression, although it increases the net melanogenesis in skin. This effect must be related rather to the increased number of melanocytes, than to any specific regulatory activity of HGF/SF towards MITF that we have detected.

In conclusion, our particularly important observation is that transgenic HGF/SF did not influence the type of pigment produced by C57BL/6 mice, nor the net production of eumelanin in the hair follicles. Eumelanin but no pheomelanin was detected in transgenic C57BL/6-HGF or C57BL/6 wild type mice although both pheomelanin and eumelanin were found in yellow mutant C57BL/6-Mc1r ^e/e^ and C57BL/6-A^y/a^ skin and hair. Thus, pheomelanin does not participate in melanomagenesis in this model. Transgenic HGF/SF did not affect the skin thickness in telogen nor in the last, postnatal stages of hair follicle morphogenesis, but it increased the number of hair follicles and the number of extrafollicular melanocytes in the dermis. No melanocytes were found in the epidermis and no melanosomes were found in epidermal keratinocytes. HGF/SF transgenic mice thus lack the epidermal melanin UV-protection found in constitutively dark human skin. We conclude that melanocytes in the HGF/SF transgenic mouse, particularly in the upper papillary dermis, are vulnerable to UVA which interacts with eumelanin but not pheomelanin to induce melanoma. Further, since HGF/SF does not affect melanogenesis, but influences the number and localization of the melanin-producing cells, this makes the C57BL/6-HGF/SF transgenic mouse an important model to study the evolution of vertebrate skin pigmentation, and skin pathology.
